# Proportional Cerebellum Size Predicts Fear Habituation in Chickens

**DOI:** 10.3389/fphys.2022.826178

**Published:** 2022-02-17

**Authors:** Diego Stingo-Hirmas, Felipe Cunha, Rita France Cardoso, Laura G. Carra, Lars Rönnegård, Dominic Wright, Rie Henriksen

**Affiliations:** ^1^IFM-Biology, Linköping University, Linköping, Sweden; ^2^School of Technology and Business Studies, Dalarna University, Falun, Sweden; ^3^Department of Animal Breeding and Genetics, Swedish University of Agricultural Sciences, Uppsala, Sweden

**Keywords:** emergence test, behavioral predictability, domestication, neural density, isotropic fractionation

## Abstract

The cerebellum has a highly conserved neural structure across species but varies widely in size. The wide variation in cerebellar size (both absolute and in proportion to the rest of the brain) among species and populations suggests that functional specialization is linked to its size. There is increasing recognition that the cerebellum contributes to cognitive processing and emotional control in addition to its role in motor coordination. However, to what extent cerebellum size reflects variation in these behavioral processes within species remains largely unknown. By using a unique intercross chicken population based on parental lines with high divergence in cerebellum size, we compared the behavior of individuals repeatedly exposed to the same fear test (emergence test) early in life and after sexual maturity (eight trials per age group) with proportional cerebellum size and cerebellum neural density. While proportional cerebellum size did not predict the initial fear response of the individuals (trial 1), it did increasingly predict adult individuals response as the trials progressed. Our results suggest that proportional cerebellum size does not necessarily predict an individual’s fear response, but rather the habituation process to a fearful stimulus. Cerebellum neuronal density did not predict fear behavior in the individuals which suggests that these effects do not result from changes in neuronal density but due to other variables linked to proportional cerebellum size which might underlie fear habituation.

## Introduction

The cerebellum, the most neuron dense brain region (Herculano-Houzel et al., 2014; Olkowicz et al., 2016), is highly conserved in its anatomy and circuitry among jawed vertebrates (Voogd and Glickstein, 1998) but varies widely in both absolute and relative sizes (Eager, 1967; Yopak et al., 2020). This variation in cerebellar size has been extensively associated with differences in motor skills across species (Iwaniuk et al., 2007, 2009; Yopak et al., 2007; Hall et al., 2013). For example, in bower building birds the complexity of these structures correlates positively with cerebellum size (Day et al., 2005). While traditionally the cerebellum has mainly been seen as a sensory motor control system (Strick et al., 2009), recent findings from neuroanatomical, neuroimaging, and behavioral studies propose a paradigm shift on this view (Schmahmann, 1991; Tedesco et al., 2011; Koziol et al., 2014; Hoche et al., 2018). By demonstrating that the cerebellar output targets several non-motor cortical areas (see review Strick et al., 2009), the range of functions associated with the cerebellar activity has been remarkably expanded, including associative learning, memory, and emotional processing (Schmahmann, 1991; Ferrucci et al., 2012; Buckner, 2013; Lupo et al., 2015; Adamaszek et al., 2017).

Fearfulness behavior is one of the many functions recently suggested to be linked with the cerebellar anatomy and activity across different vertebrate species (Yoshida and Hirano, 2010; Møller and Erritzøe, 2014; Symonds et al., 2014; Moreno-Rius, 2018; Katajamaa et al., 2021). In birds, for instance, the relative size of the cerebellum appears to predict predator-prey interactions, such as the flight initiation distance of a species (Møller and Erritzøe, 2014; Symonds et al., 2014). However, whether cerebellum size is positively or negatively correlated with fearfulness responses across species remains a matter of debate (Møller and Erritzøe, 2014; Symonds et al., 2014). At least within species, in the case of the red junglefowl (*Gallus gallus*), the size of the cerebellum is positively associated with the memory of fear-like stimuli (Katajamaa et al., 2021). When repeatedly exposed to the same fearful stimuli, junglefowl chicks with reduced fearfulness reactions (faster habituation) had larger cerebellum when adult than chicks with stronger fearfulness reactions (Katajamaa et al., 2021). To what extent, however, this behavioral pattern of habituation to fear is consistent throughout the development of the chicken, and whether the increase in cerebellum size reflects proportionally more neurons is still unknown.

Here, to test if the link between cerebellum size and fear habituation is consistent across development, we performed repeated behavioral testing in a fear inducing test (the emergence test) early in life and after sexual maturity (eight trials per age group) in a population of 36 chickens bred from a unique advanced intercross between domestic chickens (White Leghorn) and their wild ancestor, the red junglefowl. Domestic chickens have a much larger cerebellum (both absolute and proportionally to the rest of the brain, Henriksen et al., 2016) with proportionally more neurons (Racicot et al., 2021) than red junglefowl, and differ significantly in their response to fearful stimuli (Campler et al., 2009). By interbreeding these two chicken populations, we were able to generate a population with large phenotypic variation in (1) cerebellum size (both absolute and proportional to the rest of the brain) and (2) fearfulness. With this population, we could test the link between fear habituation and cerebellum size down to the neural level. Based on previous findings, we hypothesized that both young and adult chickens habituating more rapidly to the emergence tests have larger cerebellum than chickens that require more time to habituate. We also predicted that size differences in the cerebellum among chickens would reflect proportional shifts in the numbers of cerebellar neurons.

## Materials and Methods

### Animal Rearing and Housing

We raised 36 individuals from a highly advanced intercross (F18) chicken population that began with one red junglefowl male originated from Thailand (*Gallus gallus*) and three White Leghorn females (*Gallus gallus domesticus*) (Höglund et al., 2020). All individuals were group housed indoor and were kept in day-night cycles of 12/12 h until 10 weeks of age, after which they had access to an outdoor area. Perches, food, and water were provided *ad libitum*. During behavioral testing early in life the chicks were kept in groups of six per cage, while during the adult testing the individuals were kept in single cages within the same room during the testing days. At 229 days of age all individuals were culled by neck dislocation and subsequently decapitated.

#### Ethics Statement

The study was approved by the local Ethical Committee of the Swedish National Board for Laboratory Animals.

### Emergence Test

The emergence test is an adaptation of the “Hole-in-the-wall box test” (as reported by Bryan Jones and Mills, 1983) and is commonly used for assessing a general fear response in chickens (Forkman et al., 2007; Archer and Mench, 2014, 2017; Ericsson et al., 2016). We tested the latency of the birds to emerge from a box at 5 to 6 weeks of age and again after sexual maturity at 26 to 27 weeks of age. The recorded tests were used for scoring the latencies of the individuals to (a) emerge their head outside the box (head-emergence) and (b) emerge their whole body outside of the box (body-emergence).

#### Young Testing Round

All chicks were tested once per day during eight consecutive days. The chicks were individually placed in boxes of 19.5 by 39.5 by 21cm (W*L*H) with the bottom covered with absorbent paper that was replaced between trials. The box was placed up against the shorter end of the arena and had an opening of 7 cm × 17 cm toward the open arena. Eight individuals were tested simultaneously in individual arenas, and both the boxes and arenas were in complete darkness until the filming of the test started; lights were then turned on only inside the arena, leaving the box in gloom. The tests lasted until the bird had fully emerged from the box or a maximum of 5 min if the bird did not emerge. Recordings were done using video cameras connected to a computer. Tests were carried out from 9am to 12pm and the order of testing for each individual was randomized every day.

#### Sexual Mature Testing Round

Half of the individuals (*n* = 18) were tested during 4 consecutive days the first week of testing and the other half (*n* = 18) was tested during 4 consecutive days during the second week of testing. Each individual was tested twice a day (morning and afternoon). The chickens were placed in a box (58L:35W:46H) and let to acclimatize for 30 s. A light was then turned on outside of the box, and a hatch (17 by 21 cm) was opened. The hatch gave access to an arena of 2.79 m long and 93 m wide.

Both the box and the arena floors were covered with a bedding of wood shavings that was cleaned from feathers and feces after each test. Three individuals were tested simultaneously in separate arenas of identical conditions, with the test lasting until the chicken emerged the box or a maximum of 5 min if the bird did not emerge. Tests were recorded using cameras GoPro Black 7© (2021 GoPro Inc.) and were carried out from 9am to 11:30 (morning), and a second time from 12pm to 14:30 (afternoon). The order in which individuals were tested was maintained between mornings and afternoons but was randomized across the days.

### Brain Measurements

For all the individuals, brains were extracted just after the culling, and dissected into cerebrum (telencephalon), cerebellum, optic tectum, and brainstem region (thalamus, remaining midbrain, and hindbrain) (sensu Henriksen et al., 2016). The brain regions were immediately weighed after dissection and then the right cerebral hemisphere as well as the right optic tectum and the right half of the cerebellum (cut down the vermis, and each half weighed) was fixed in 4% paraformaldehyde in 0.1 M phosphate buffer for at least 72 h. The left-half of the cerebrum, optic tectum, cerebellum as well as the brainstem regions were flash-frozen in liquid nitrogen and stored at −80°C for later studies. For the cerebellum, we estimated numbers of total cells, neurons, and non-neuronal cells following the isotropic fractionator technique (Herculano-Houzel and Lent, 2005). The right half of the cerebellum was analyzed for cell counting and the numbers obtained were multiplied by 2. As described elsewhere, the isotropic fractionator consists in mechanically dissociating the brain tissue (e.g., cerebellum) in 40 mM sodium citrate with 1% Triton X-100 using Tenbroeck tissue homogenizers (Herculano-Houzel and Lent, 2005; Olkowicz et al., 2016). Once the brain is transformed into a suspension of free cell nuclei with a defined volume and kept homogeneous by agitation, the total number of cells was estimated by using fluorescent DNA marker 4′,6-Diamidine-2′-phenylindole dihydrochloride (DAPI). At least four aliquots (10 μL) per individual were sampled and counted using Neubauer improved chamber under a fluorescent Nikon eclipse 80i microscope at 400× magnification. For the cell counts, the coefficients of variation among the four aliquots were lower than 0.15. To determine the proportion of neurons across our samples, we used immunocytochemical detection of neuronal nuclear antigen NeuN, expressed in the nuclei of most neuronal populations within the brain (Mullen et al., 1992). Although NeuN is not expressed by Purkinje cells (Mullen et al., 1992), these cells do not represent the largest fraction of cells in the cerebellum (Cunha et al., 2020, 2021), and therefore not sampling them should not be a major issue for our comparative dataset. We used mouse monoclonal antibody anti-NeuN 488 AlexaFluor conjugated (1:300 in phosphate-buffered saline; clone A60, Chemicon; MAB377X), incubated at 10°C overnight. A minimum of 500 nuclei was counted to estimate the proportion of neurons (immunolabeled in Dapi and NeuN +) in the sample. The number of non-neuronal cells in the cerebellum was obtained through subtraction.

### Statistical Analysis

**FIGURE 1:**
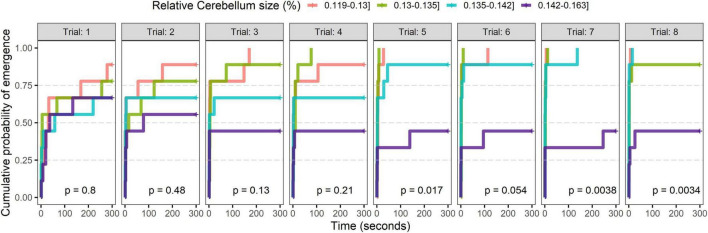
Kaplan-Meier survival curves for adult individuals showing the effect of proportional cerebellum size (%cerebellum) on habituation during repeated trials (eight trials in total) in an emergence test. Proportional cerebellum size is divided in quartiles with equal number of individuals in each group. Significance for differences in survival curves between groups are calculated with a log-rank test represented by the p-value in each graphic.

All analyses were performed using R version 4.1.1 (R Core Team, 2021). Data on the time taken for the chickens to head-emerge or body-emerge during the 16 trials (8 trials early in life and eight trials after sexual maturity) included multiple censored observations, in which individuals failed to emerge. Cox proportional hazards models were therefore used to analyze the effect of cerebellum size and other explanatory variables on the time taken to emerge. Cox proportional hazards models were built with the package “survival” (Therneau, 2021). Covariates of the four brain regions size [both absolute (g) and in proportion (%) to total brain size (g)], trial number, sex, and cerebellar neuronal density (CND) were tested using the *coxph* function. Cerebellar neural number and absolute mass are strongly correlated (see Supplementary Figure 2) but the correlation varies between individuals and therefore both variables were included in the model. The data was clustered by individual to account for correlations among repeated observations of the same bird and inter-individual variation in initial fear response (trial 1). In Cox proportional hazard models, estimated coefficients are log hazard ratios. Hence, coefficients that are > 0 correspond to hazard ratios > 1 and increased risk of emergence. For class variables it is an increased risk compared to the reference class, whereas for continuous variables the risk increases with increased values of the covariate. Behavioral observations were grouped by both the day (1 to 8 for young, 1 to 4 for mature) and the number of tests (1 test per day for chicks and 2 tests within a day for chickens). For observations where the animal did not head- or body-emerge, a maximum value of 300 s was given. The time to head-emerge and body-emerge was highly correlated across all observations (Spearman’s correlation coefficient = 0.956, *P* < 0.001). Because of this strong relationship between head- and body emergence, we chose to use head emergence as our main response variable for the proportional hazards models, as it is deemed to fairly represent the results with a reduced number of censored observations (i.e., chickens that did not head-emerge) in the data. Since behavioral testing was done during an eight-day period for the chickens early in life and during a four-day period when they reach sexual maturity, the cox proportional hazards models were fitted separately for each age. For further visualization of the results, all individuals were divided into four quartiles of nine individuals in each age group based on proportional cerebellum size, and Kaplan-Meier curves (Kassambara et al., 2021) were plotted for each trial (see Figure 1), and log-rank tests were used to estimate the significance of the differences between the curves. Because Kaplan-Meier estimates commonly represent *y* as the probability of survival at time *t* (chance of the event not occurring), survival curves in the graphics were transformed such that *f*(*y*) = 1−*y*. Therefore, Figure 1 represent the likelihood of the individual emerging at time t.

## Results

The between-individual variation in time taken for the head to emerge during the emergence test ranged from 16.7% on the first day to 61.1% on the last day when tested early in life, and from 75 to 83.3% when tested after sexual maturity. For the adult individuals, the time of day (morning vs. afternoon) had no effect on the latency for head emergence, and this variable was therefore removed from the model, making the final model used for each age group the same. There was a significant effect of proportional cerebellum size on the latency to head emergence in adult chickens during the eight trials with increasing significance as the trials progress (see Table 1). Absolute cerebellum size as well as the size (absolute and proportional) of the other three brain regions had no significant effect on latency to emerge (*p* > 0.05) in adult chickens and was therefore removed from the final model.

**TABLE 1:** Adult test rounds: Hazard ratios of proportional cerebellum size (%cerebellum), trial number, and the interaction effect between both variables (denoted by “:”), when controlling for sex, for the latency to head-emerge.

Covariate	Coefficient	Hazard ratio	Robust se	*p*-value
Sex (female)	0.15	1.16	0.39	0.70
%Cerebellum size	–0.17	0.85	0.20	0.41
Trial 2	2.01	7.47	3.50	0.57
Trial 3	5.11	165.21	3.31	0.12
Trial 4	5.09	162.63	3.01	**0.09**
Trial 5	5.56	260.07	2.92	**0.06**
Trial 6	7.82	2492.73	3.26	**0.02**
Trial 7	9.40	12080.87	3.14	**0.003**
Trial 8	9.03	8327.15	2.93	**0.002**
%Cerebellum: Trial 2	–0.14	0.87	0.26	0.59
%Cerebellum: Trial 3	–0.36	0.70	0.25	0.15
%Cerebellum: Trial 4	–0.35	0.70	0.22	0.11
%Cerebellum: Trial 5	–0.37	0.69	0.22	**0.09**
%Cerebellum: Trial 6	–0.52	0.59	0.25	**0.03**
%Cerebellum: Trial 7	–0.62	0.54	0.23	**0.007**
%Cerebellum: Trial 8	–0.60	0.55	0.22	**0.007**

*Trial 1 used as reference level in Cox proportional hazards model.*

*Bold indicates statistical significance.*

The latency to emerge when the individuals were tested early in life was not significantly affected by the interaction between trial number and cerebellum size (both absolute and proportional, *p* < 0.05), and the interactions were therefore removed from the final model. Similarly, none of the three other brain regions sizes (absolute and proportional) had any significant effects on latency to emerge early in life (*p* > 0.05) and were therefore removed from the final model. Latency for head emergence was, however, significantly affected by trial number early in life, with latency being significantly different on all trials compared to trial 1 (see Table 2). Sex had no effect on latency to head-emerge neither in young (*P* = 0.99) or adult birds (*P* = 0.70), and neither did cerebellum neural density (adult: *p* = 0.93, young: *p* = 0.313); the later variable was therefore removed from the final models (see Tables 1, 2). The Kaplan-Meier survival curves for all adult individuals divided into quartiles based on proportional cerebellum size (Figure 1) showed that proportional cerebellum is negatively correlated with an individual’s likelihood to emerge as the trials progress in adult individuals (see Figure 1).

**TABLE 2:** Young test rounds: Hazard ratios for latency to head-emerge, as affected by the number of trials.

Covariate	Coefficient	Hazard ratio	robust se	*p*-value
Sex (female)	5 × 10^–4^	1.00	0.35	0.999
%Cerebellum size	0.21	1.23	0.16	0.2
Trial 2	0.91	2.48	0.33	**0.006**
Trial 3	1.23	3.43	0.40	**0.002**
Trial 4	1.50	4.49	0.42	**0.0003**
Trial 5	1.43	4.17	0.48	**0.003**
Trial 6	1.80	6.07	0.47	**0.0001**
Trial 7	1.89	6.59	0.49	**0.0001**
Trial 8	1.71	5.54	0.45	**0.0002**

*Trial 1 used as reference level in Cox proportional hazards model. Bold indicates statistical significance.*

## Discussion

We find that proportional cerebellum size is linked to the behavioral response of adult chickens tested repeatedly in an emergence test. While proportional cerebellum failed to predict fearfulness during the initial trials, the proportional size of this brain region predicted fearfulness as the trials progressed. The correlation was unaffected by sex and no other brain region (proportional or absolute) correlated with fearfulness. This indicates that, while proportional cerebellum size is not linked to a chicken’s initial fear response in an anxiety test, it is linked to the change in behavior that occurs when an individual is repeatedly exposed to the same anxiety stimuli. In Figure 1 we can see that although most individuals seem more likely to emerge faster from the emergence box as the trials progress, adult individuals with the largest proportional cerebellum (upper 25%) tended to stay slightly longer in the emergence box as the trials progressed. When looking at the behavior of these individuals early in life, there was no significant correlation between time to emerge and adult proportional cerebellum or any other brain measurement.

Our findings are comparable to previous finding on red junglefowl in which adult cerebellum size, but no other brain region, correlated with habituation to fear measured early in life (Katajamaa et al., 2021). In this study the authors found that chicks with the largest proportional cerebellum as adult seem to emerge sooner (we found a similar non-significant correlation for the chicks in our study, see Supplementary Figure 1). Yet, as we can see from our study this pattern is reversed when testing fear habituation in adult chickens. While most of the adult individuals appear to habituate and thereby reduce their fear response in the emergence test, the individuals with the largest proportional cerebellum seem to dishabituate (sensitize) by increasing their fear response as the trials progress. Habituation is a form of simple learning in which the magnitude of the response to a specific stimulus decreases with repeated exposure to that stimulus (Rankin et al., 2009). It is believed that habituation allows animals to filter out irrelevant stimuli and focus selectively on important stimuli (Rankin et al., 2009). According to the *dual-process theory*, habituation and dishabituation (sensitization) processes act independently and then sum together to produce the observed behavioral effects of repeated stimulation (Groves and Thompson, 1970), indicating that slightly different neural pathways might be involved in these two behavioral processes. Although habituation is well studied across species, very little is known about the neural mechanisms underlying this behavioral response (Rankin et al., 2009). As one of the key functions of the cerebellum is to identify and store sequences of actions (Popa and Ebner, 2019), the cerebellum’s involvement in fear response, reported in this study (and the study by Katajamaa et al., 2021) could be due to its ability to predict future outcome when exposed to the same stimuli multiple times (*sequence detection hypothesis* by Leggio and Molinari, 2015).

It is interesting that the adult proportional size of the cerebellum to some extent predicts habituation behavior of chickens both early in life and after sexual maturity, but it is baffling as to why the link is reversed during development. The size of the cerebellum, as well as other major brain regions, changes in absolute as well as proportional size during post-hatch development in chickens (Henriksen et al., 2016). For example, the cerebellum exhibits its largest proportional size in the brain around four weeks of age in domestic chickens and red junglefowl and has a much smaller proportional size both before and after this age (Henriksen et al., 2016). The adult chicken brain is therefore not just a scaled-up version of the chick brain, which makes it difficult to correlate adult cerebellum size with chick behavior and to predict the brain composition of these chicks early in life. Furthermore, although the proportional size of all major brain regions follows the same growth trajectories in red junglefowl and domestic chickens, the proportional size of these brain regions still shows substantial variation at any given age between wild and domestic chickens (Henriksen et al., 2016). Since the population used in our study was an intercross between red junglefowl and domestic chickens this further increases the range of possible variation in brain composition between these individuals early in life.

Our study is not the first to demonstrate a link between the cerebellum and the behavioral process of habituation. Previous brain positron emission tomography (PET) studies in humans (Timmann et al., 1998) and lesion studies in rodents (Leaton and Supple, 1991) have reported the cerebellum to be involved in the habituation of individuals startle response to fearful acoustic stimuli, but our results are the first to indicate that the link between adult cerebellum and habituation could be influenced by the proportional size of this brain region. To further explore the correlation between fear habituation and the functional significance of cerebellum size we measured neural density in the cerebellum. Neural density, however, did not predict fear habituation, demonstrating that the proportional size of the cerebellum is a better predictor for habituation behavior in chickens than neural number and density. This finding indicates that factors other than neural density, linked to proportional cerebellum size, could be responsible for affecting the degree of habituation. For example, our results suggest that the observed effects are not simply due to proportionally larger cerebellum having greater or lesser neuronal density, but rather is representative of different mechanisms that are linked with proportional size. The advanced intercross population used in this study was based on red junglefowl and domestic White Leghorn chickens. We have previously demonstrated that the cerebellum has increased during domestication, both proportional to the brain and in absolute size (Henriksen et al., 2016). Additionally, this increase in cerebellar size in domestic chickens is associated with an increase in the foliation of the cerebellum, granule cell layer size, granule cell size and number, and Purkinje cell number (Racicot et al., 2021). The intercross population used in this study therefore have the potential to vary in all these brain parameters. Two limitations to the isotropic fractionation technique used in this study is that it does not include Purkinje cells in the cell counting sample, nor can it be used to assess the size of the different neurons in the cerebellum. Additionally, it was outside the scope of this study to measure cerebellar foliation. We can therefore only speculate about the significance of these variables.

Our finding that increased proportional cerebellum size is linked to dishabituation in adult chickens and thereby higher fear response in a fear inducing emergence test, is in accordance with a previous study by Møller and Erritzøe (2014). Here the authors demonstrate that across bird species increased proportional cerebellum is linked to increased antipredator response, and thereby fear as this behavior is generally defined as a reaction to the perception of danger (Boissy et al., 1998). This result, combined with ours suggests that both within and among species the proportional size of the cerebellum is linked to fear response in birds, but as suggested by our findings (and previous finding by Katajamaa et al., 2021) this link might have more to do with the ability of the cerebellum to store sequences of actions and thereby the ability of this brain region to modify the behavioral fear response when repeatedly exposed to the same type of fear inducing stimuli.

## Data Availability Statement

The raw data supporting the conclusions of this article will be made available by the authors, without undue reservation.

## Ethics Statement

The animal study was reviewed and approved by the Local Ethical Committee of the Swedish National Board for Laboratory Animals.

## Author Contributions

RH and DW conceived the study. DS-H, FC, RC, LC, DW, and RH collected data. DS-H and LR performed the analysis. DS-H, FC, and RH wrote the initial manuscript. All authors revised and contributed to the initial and final draft.

## Conflict of Interest

The authors declare that the research was conducted in the absence of any commercial or financial relationships that could be construed as a potential conflict of interest.

## Publisher’s Note

All claims expressed in this article are solely those of the authors and do not necessarily represent those of their affiliated organizations, or those of the publisher, the editors and the reviewers. Any product that may be evaluated in this article, or claim that may be made by its manufacturer, is not guaranteed or endorsed by the publisher.
