# Supplementary material for: Proportional Cerebellum Size Predicts Fear Habituation in Chickens
Source: Front Physiol. 2022 Feb 17;13:826178. doi: 10.3389/fphys.2022.826178 (PMC8891606; doi:10.3389/fphys.2022.826178)
Supplement: Supplementary file 1 [file Data_Sheet_1.PDF]

Supplementary figures

(Proportional cerebellum size predicts fear habituation in chickens)

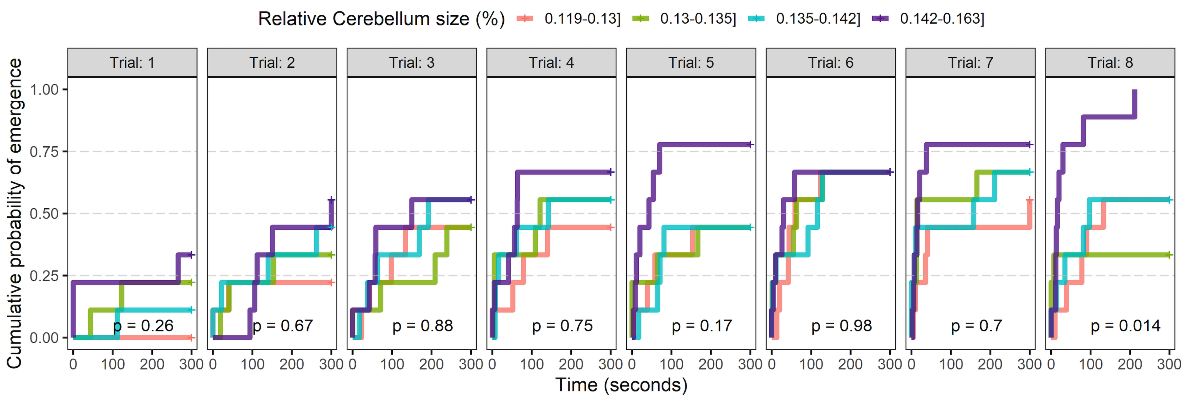

Figure 1. Kaplan-Meier survival curves for adult individuals showing the effect of proportional cerebellum size (%cerebellum) on habituation during repeated trials (eight trials in total) in an emergence test. Proportional cerebellum size is divided in quartiles with equal number of individuals in each group. Significance for differences in survival curves between groups are calculated with a log-rank test represented by the p-value in each graphic

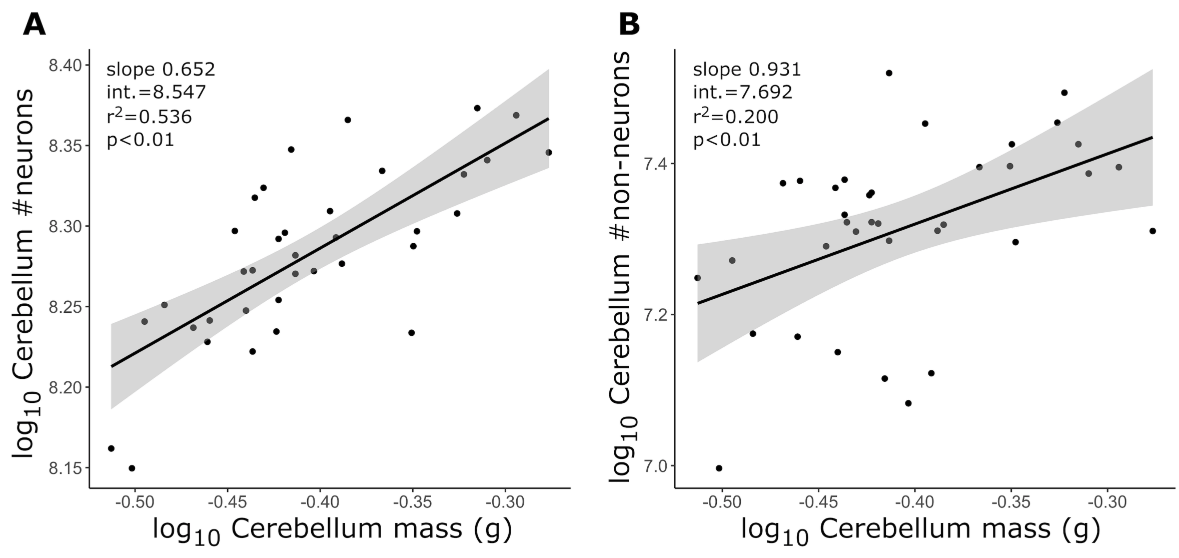

Figure 2. Correlations between cerebellum mass (g) and A) neural number and B) non-neural number.
